# Implementing an Intervention Program During the COVID-19 Pandemic: Challenges and Successes

**DOI:** 10.1093/geroni/igab046.1007

**Published:** 2021-12-17

**Authors:** Nancy Mendoza, Cherrie Park

**Affiliations:** 1 The Ohio State University, The Ohio State University, Ohio, United States; 2 The Ohio State University, Columbus, Ohio, United States

## Abstract

During the COVID-19 pandemic, the implementation of intervention programs for grandfamilies are facing multiple challenges. In this paper, we will present some of the challenges and successes of introducing the GRANDcares Plus Project (GRANDc+) during the COVID-19 pandemic. As an intervention program, GRANDc+ has demonstrated positive outcomes for grandfamilies, such as increased satisfaction with life, knowledge of services, self-care practices, and supportive social networks. Due to the pandemic, the implementation of GRANDc+ has been met with many challenges including, training of facilitators, following CDC’s COVID-19 guidelines/recommendations, and considering grandfamilies needs, concerns and safety. The pandemic has and continues to have detrimental effects on grandfamilies; this makes it more vital than ever to support grandfamilies through interventions like GRANDc+, despite what challenges we may face. Our presentation will provide insights into identifying, managing, and overcoming the challenges of implementing interventions during the COVID-19 pandemic.

